# Spread of *mcr-1*–Driven Colistin Resistance on Hospital Surfaces, Italy

**DOI:** 10.3201/eid2409.171386

**Published:** 2018-09

**Authors:** Elisabetta Caselli, Maria D’Accolti, Irene Soffritti, Micol Piffanelli, Sante Mazzacane

**Affiliations:** Università Degli Studi di Ferrara, Ferrara, Italy

**Keywords:** Colistin resistance, surface contamination, hospitals, Italy, bacteria, antimicrobial resistance, Acinetobacter Iwoffii, Citrobacter freundii, Enterobacter cloacae, Enterobacter agglomerans, Escherichia coli, Klebsiella pneumoniae, Klebsiella oxytoca, Pseudomonas aeruginosa, Pseudomonas putida, mcr-1

## Abstract

Plasmid-mediated colistin resistance driven by the *mcr-1* gene is of great clinical concern. Its diffusion in the hospital environment is unknown. We detected *mcr-1*–driven resistance in 8.3% of *Enterobacteriaceae* isolates from hospital surfaces in Italy, which might represent a reservoir of threatening nosocomial pathogens.

The rapid and continuous growth of drug resistances is of global concern and one of the most severe threats for human health. Among those detected in recent years, a plasmid-mediated colistin resistance, driven by the *mcr-1* gene (*1*), represents a serious clinical concern because colistin was considered a last-resort drug against multidrug resistant (MDR) gram-negative bacteria.

Since its original isolation in an *Escherichia coli* strain in China in 2016, the *mcr-1* gene has been detected almost globally in ≈10% of animal isolates (*2*) and in 0.1%–2% of human isolates (*3*), suggesting that this plasmid-mediated resistance spread efficiently from animals (where colistin has been used for years as a therapeutic drug or food supplement) to humans through horizontal gene transfer. Furthermore, the *mcr-1* gene was found in different gram-negative bacteria, including *Klebsiella pneumoniae*, *Enterobacter*, *Salmonella* (*1*,*4*,*5*), and recently *Citrobacter* (*6*). The emergence of *mcr-1* in clinical *Enterobacteriaceae* isolates appears particularly alarming because it frequently occurs in MDR strains, further limiting current treatment options for lethal infections sustained by carbapenem-resistant *Enterobacteriaceae*.

In Italy, *mcr-1*–driven colistin resistance was first reported in an *E. coli* strain in 2016 (*7*). However, colistin resistance already had been reported previously in carbapenem-resistant *Enterobacteriaceae* from different peripheral laboratories in Italy (*8*).

The *mcr-1* gene has been detected in infected persons, but its epidemiology is poorly described, and data are lacking about its presence in the microbial population that persistently contaminates hospital environments. Surface contamination is known to contribute to the onset of healthcare-associated infections, which are often sustained by MDR or even pan–drug-resistant strains. Thus, based on the need for information about this aspect, we aimed to determine the diffusion of *mcr-1*–driven colistin resistance in the hospital environment. 

We searched for the presence of *mcr-1* gene in our library of 300 *Enterobacteriaceae* samples collected from the surfaces of 8 hospitals in Italy during 2016–2017. Surface samples were collected from 3 points in hospital rooms (floor, bed footboard, and sink) as previously described (*9*), then grown in MacConkey broth for 48 h at 37°C to amplify the *Enterobacteriaceae* population. An aliquot of grown bacteria was frozen in 50% sterile glycerol for subsequent identification and functional studies. The remaining bacterial suspension was used for total DNA extraction (UCP-Pathogen Mini Kit; QIAGEN, Hilden, Germany) and analyzed for *mcr-1* gene presence by nested PCR. We conducted first-round amplifications as previously described (*1*); nested PCR amplification was carried out using the following primers and conditions: CLRn-F (5′-AAA CCT ATC CCA TCG CGG AC-3′) and CLRn-R (5′-CCG CGC CCA TGA TTA ATA GC-3′), for 35 cycles at 57°C, originating a 147-bp amplification product, subsequently confirmed by sequence analysis. Plasmid pBAD24::mcr-1 (*3*) was used as a positive control. We also conducted a universal panbacterial PCR as a control of DNA amplification (*9*). Whole-genome sequence and *mcr-1* location were not analyzed here and might deserve future study.

Of 300 *Enterobacteriaceae* isolated from hospital surfaces, 25 (8.3%) harbored the *mcr-1* gene. All positive samples were culturally isolated on MacConkey agar plates. We identified presumptive positive isolates at the species level by biochemical typization (API-20E) and Vitek-2 system (BioMérieux, Florence, Italy) and tested them for drug susceptibility by disc diffusion (Entero1 Multodisc; Liofilchem,Teramo, Italy) and broth microdilution (SensiTest Colistin, Liofilchem).

Identification results indicated that different species harbored the *mcr-1* gene, including *K. pneumoniae*, *K.* *oxytoca*, *E. coli*, *Acinetobacter Iwoffii*, *Enterobacter cloacae*, *E.* *agglomerans*, *Citrobacter freundii*, *Pseudomonas aeruginosa*, and *P. putida* (Table). These results suggest that this gene is silently spreading to many gram-negative bacteria responsible for infections in clinical settings. 

**Table:** Antimicrobial susceptibility of the *mcr-1*–carrying bacterial isolates from hospital surfaces, Italy*

Bacteria	No. isolates	Drug-resistant isolates, % (MIC, mg/L)								
F	AK	ATM	TZP	C	SXT	NET	CTX	Col-R
*Acinetobacter Iwoffii*	4	50	25	50	50	25	50	25	25	7 (4–8)
*Citrobacter freundii*	1	0	0	0	0	100	0	0	0	4
*Enterobacter cloacae*	3	100	100	33.3	100	33.3	33.3	100	33.3	16
*Enterobacter agglomerans*	3	100	0	0	100	0	0	100	0	5.3 (4–8)
*Escherichia coli*	4	100	50	25	100	0	25	100	50	10 (8–16)
*Klebsiella pneumoniae*	6	100	100	33.3	66.6	66.6	0	100	66.6	13.3 (8–16)
*K. oxytoca*	2	100	100	0	0	0	0	100	0	16
*Pseudomonas aeruginosa*	1	0	100	0	100	100	0	100	100	4
*P. putida*	1	0	100	0	0	100	0	100	0	8

*AK, amikacin 30 μg; ATM, aztreonam 30 μg; C, chloramphenicol 30 μg; Col-R, colistin resistant; CTX, cefotaxime 5 μg; F, nitrofurantoin 100 μg; NET, netilmicin 10 μg; SXT, trimethoprim/sulfamethoxazole 25 μg; TZP, piperacillin/tazobactam 36 μg.

All *mcr-1*–carrying isolates were colistin resistant by microdilution test (MIC 4 mg/L to >16 mg/L). In addition, as judged by the results obtained by the disc-diffusion method, all colistin-resistant isolates were resistant to >2 antimicrobial drugs among those effective against *Enterobacteriaceae*, exhibiting a MDR phenotype.

Our data show that *mcr-1*–carrying *Enterobacteriaceae* can be detected on hospital surfaces with higher frequency than in clinical isolates, indicating that this plasmid has the ability to spread, not only in vitro (*1*), in key human pathogens. Persistent surface contamination in hospitals might thus favor colistin resistance spread among gram-negative bacteria, perhaps helped by selective pressure exerted by some antiseptics (i.e., chlorhexidine) (*10*). Although this finding might represent a potential reservoir of threatening nosocomial pathogens and favor their diffusion in hospitalized patients, currently no specific monitoring exists to control it. Thus, we suggest that surveillance for *mcr-1*–driven colistin resistance might include not only clinical samples but also environmental analyses and all clinically relevant gram-negative species to control and counteract the increase of untreatable infections.
